# Mitochondrial Hijacking and MicroRNA Crosstalk: Cancer Stem Cell-Mediated Immune Evasion and Metabolic Plasticity in the Tumor Microenvironment

**DOI:** 10.3390/cancers18101611

**Published:** 2026-05-15

**Authors:** Maziar Ashrafian Bonab, Shahrzad Salehi, Amirreza Aghababaie, Ali Amini, Hoda Alizadeh, Babak Behnam

**Affiliations:** 1 School of Geography and Natural Sciences, Faculty of Science and Environment, Northumbria University, Newcastle NE1 8ST, UK; 2 Medical Genetics Network (MeGeNe), Universal Scientific Education and Research Network (USERN), Tehran 14197331, Iran; 3 Biology Department, American University, Washington, DC 20016, USA; 4 Department of Biosciences, College of Health, Medicine and Life Sciences, Brunel University, Kingston Campus, Uxbridge, London UB8 3PH, UK; 5 Floret Center for Advanced Genomics and Bioinformatics Research, Lagos 100282, Nigeria; 6 Avicenna Biotech Research, Clarksburg, MD 20871, USA

**Keywords:** tumor microenvironment, cancer stem cells, mitochondrial hijacking, microRNA crosstalk, immune evasion, metabolic plasticity

## Abstract

The tumor microenvironment (TME) is a highly adaptive and heterogeneous niche in which cancer stem cells (CSCs) promote immune evasion, metastatic dissemination, and therapy resistance. Among the mechanisms that support this phenotype, mitochondrial hijacking has emerged as a central strategy through which CSCs reprogram immune and stromal cells to favor tumor progression. This review synthesizes current evidence on how CSCs exploit mitochondrial transfer, particularly via tunneling nanotubes (TNTs) and extracellular vesicles (EVs), to impair antitumor immunity and remodel the metastatic niche. CSCs display marked metabolic plasticity, shifting between glycolysis and oxidative phosphorylation (OXPHOS) in response to environmental stress. They exploit this adaptability by transferring mitochondria and mitochondrial components to recipient cells, including tumor-associated macrophages (TAMs) and cytotoxic T cells, thereby disrupting ATP production, increasing oxidative stress, and skewing immune polarization. This mitochondrial hijacking contributes to an immunosuppressive milieu, stabilizes HIF-1α, and enhances PD-L1 expression, ultimately weakening T-cell activity and reinforcing CSC survival. EVs add another layer of regulation by transporting bioactive cargo, including oncogenic microRNAs (miRNAs) and mitomiRs such as miR-21, miR-210, and miR-34a. These molecules modulate mitochondrial gene expression, reshape immune signaling, and reinforce CSC phenotypes through autocrine and paracrine loops. Single-cell and spatial transcriptomic approaches have further revealed metabolic heterogeneity within CSC–immune synapses, identifying “metabolic hotspots” associated with profound immune dysfunction. Therapeutic strategies targeting OXPHOS, EV biogenesis, and miRNA activity are therefore being explored. In parallel, mitochondria-associated proteins such as TSGA10 may also contribute to CSC-driven immunometabolism regulation and deserve further investigation. Targeting downstream heterogeneity is like cutting the branches of a weed. Targeting the upstream mechanisms of mitochondrial hijacking and miRNA crosstalk aims to destroy the root (CSC plasticity) that generates the heterogeneity and drives therapy resistance in the first place. This review highlights mitochondrial hijacking and miRNA-mediated reprogramming as central determinants of CSC-driven immune escape and proposes a framework for precision interventions targeting CSC–immune interactions in metastatic cancer.

## Simple Summary

Cancer stem cells (CSCs) are a dangerous part of tumors because they help the cancer spread, avoid the immune system, and resist treatment. One way they do this is by “stealing” or transferring their own mitochondria (the energy-producing parts of cells) into nearby immune cells, which weakens those immune cells. They also release tiny particles (extracellular vesicles) carrying microRNAs that further disrupt immune function. The result is a highly protective environment for cancer. Instead of just fighting the many different cancer cells that arise (the “branches”), future treatments should target the root cause: the way CSCs hijack mitochondria and use microRNAs to reprogram the immune system.

## 1. Introduction

### Conceptual Framework: Integrating Mitochondrial Transfer and miRNA Crosstalk as a Unified CSC Survival Axis

Prior reviews have separately addressed cancer stem cell (CSC) metabolism [1,2], intercellular mitochondrial transfer [3,4], or miRNA-mediated immune modulation [5,6]. However, the field has lacked an integrated framework that explains how CSCs coordinately exploit both physical organelle exchange and nucleic acid-based signaling to enforce immune evasion and metabolic dominance. Multiple pathways mediate this transfer: tunneling nanotubes (TNTs), extracellular vesicles, or direct cell–cell contact (gap junctions) [1,7,8]. This review fills that gap by proposing that CSCs operate through a dual-layer communication system: a “hardware” layer involving direct mitochondrial transfer (via tunneling nanotubes and extracellular vesicles) and a “software” layer comprising miRNA-enriched extracellular vesicles (EVs) that reprogram recipient cell gene expression [3,5]. True mitomiRs are encoded by the mitochondrial genome (mtDNA) and regulate mitochondrial transcription nuclear-encoded miRNAs that target mitochondrial transcripts represent a distinct class. Most ‘mitomiRs’ cited in the literature (miR-21, miR-210, miR-34a) are nuclear-encoded but function via mitochondrial localization sequences or regulate mitochondrial proteins indirectly.

Unlike prior work that treats mitochondrial hijacking and miRNA crosstalk as parallel or unrelated processes, we argue that these two mechanisms are functionally interdependent and together establish a self-reinforcing CSC survival axis. Specifically, CSC-derived mitochondria delivered to immune cells not only compromise bioenergetic capacity [9,10] but may also alter the intracellular availability of key metabolic substrates (e.g., Acetyl-CoA), while co-transferred miRNAs such as miR-21, miR-210, and miR-34a target mitochondrial transcripts and oxidative phosphorylation (OXPHOS) genes [11,12,13]. This integration provides a mechanistic explanation for the persistent immune dysfunction observed in the tumor microenvironment (TME), even when the primary tumor burden is reduced, and highlights why targeting only one arm (e.g., OXPHOS inhibition without blocking miRNA transfer) is unlikely to achieve durable responses [14,15].

The unique perspective of this review, therefore, is the conceptual unification of CSC-driven mitochondrial usurpation and miRNA-mediated metabolic reprogramming as a single, hierarchical control system. We systematically dissect the molecular components of this axis, drawing on recent single-cell and spatial transcriptomic insights that reveal “metabolic hotspots” where CSCs and immune cells engage in dysfunctional synapses [16,17,18]. We then evaluate current therapeutic strategies aimed at disrupting this axis, including OXPHOS inhibitors, EV biogenesis blockers, and miRNA antagonists [19,20], and discuss translational barriers that must be overcome. Finally, we propose a precision intervention framework that simultaneously targets both the “hardware” and “software” of CSC–immune communication. By framing these processes as a unified survival strategy, we aim to provide a roadmap for future research and for the development of combination therapies that attack the root of CSC plasticity, the mitochondrial and miRNA regulatory networks within CSCs, rather than its downstream heterogeneous manifestations [21,22].

## 2. CSC Metabolic Plasticity

### 2.1. CSCs in the Tumor Microenvironment and Metabolic Heterogeneity

Cancer stem cells (CSCs; also known as tumor-initiating stem cells) are a subpopulation of undifferentiated, highly plastic, self-renewing cells within the tumor bulk that drive tumor initiation, progression, metastasis, relapse, and resistance to therapy [23]. CSCs use molecular tools to actively maintain cellular heterogeneity and state-switching (plasticity) and have been identified across a wide range of malignancies, including breast cancer, colon cancer, glioblastoma, ovarian cancer, melanoma, pancreatic cancer, and liver cancer. Relative to non-CSCs, they often exhibit increased mitochondrial mass, elevated mitochondrial membrane potential (Δψm), and higher basal reactive oxygen species (ROS) levels. This metabolic heterogeneity among CSCs, including variable mitochondrial mass and Δψm, is increasingly recognized as a determinant of immune polarization; standardized nomenclature for mitochondrial transfer and transplantation, as recently proposed, is essential to accurately classify these processes and compare their immunomodulatory effects across studies [24].

CSCs may arise from transformed normal stem or progenitor cells, or from dedifferentiated mature cancer cells [25]. In dynamic models of tumor evolution, non-CSCs can acquire stem-like features in response to microenvironmental cues such as hypoxia and inflammatory cytokines, as well as signaling pathways including Wnt/β-catenin, Notch, and Hedgehog. This plasticity is supported by epithelial–mesenchymal transition (EMT) programs involving factors such as TGF-β and ZEB1 and may be further enhanced by mechanisms such as cell fusion [26].

**Figure 1:**
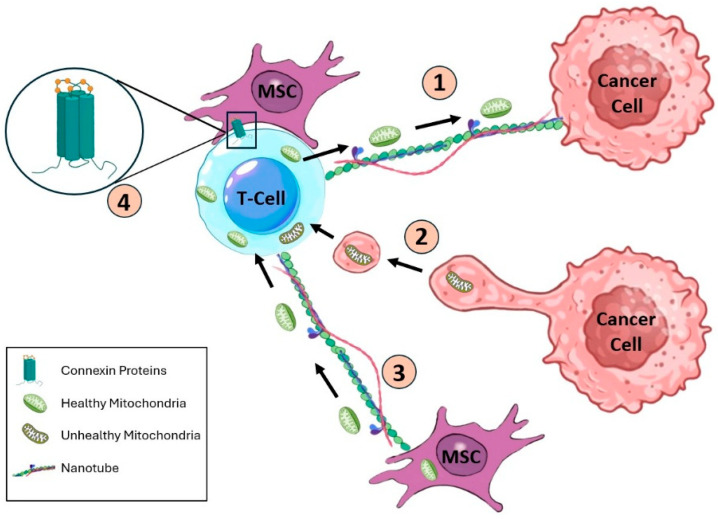
Mechanisms of intercellular mitochondrial transfer in the TME (based on Chun et al. 2025 [27]). Cancer cells (red) hijack mitochondria from T cells (blue) via tunneling nanotubes or EVs, while MSCs (pink) can donate healthy mitochondria to revive T cells. Gap junctions (Cx43) also allow direct transfer. **1**—MSC **2**—T-cell **3**—Cancer cell containing unhealthy mitochondria **4**—Nanotube connecting the MSC to the cancer cell. **Arrow:** Transfer of healthy mitochondria from the MSC (via the nanotube) into the cancer cell. Connexin proteins are shown at the interface between the cancer cell and the T-cell.

Mesenchymal stem cells (MSCs) also prominently shape the TME by promoting angiogenesis, immunosuppression, and CSC enrichment. Through secretion of factors such as IL-6, IL-8, and PGE_2_, together with the release of EVs, MSCs help maintain CSC stemness and contribute to therapy resistance and metastatic recurrence in cancers including gastric, colon, prostate, and breast malignancies [1]. This crosstalk positions the MSC–CSC interaction as a key therapeutic target. As summarized in Figure 1, MSCs interact extensively with CSCs via paracrine signaling and EVs, thereby supporting CSC maintenance and immune evasion [1].

### 2.2. Metabolic Plasticity of CSCs: Glycolysis vs. OXPHOS

The metabolic plasticity of CSCs is a direct outcome of the mitochondrial hijacking mechanisms detailed later in this manuscript, orchestrated by CSCs to control their own fate and the fate of the tumor ecosystem and microenvironment. CSCs can flexibly switch between glycolysis and oxidative phosphorylation (OXPHOS) in response to environmental cues [2]. Unlike many differentiated tumor cells, CSCs may rely on either glycolysis or mitochondrial respiration depending on context. Particularly, “CSCs show a distinct metabolic phenotype that, depending on the cancer type, can be highly glycolytic or OXPHOS-dependent” [28]. Mitochondria are thus central to CSCs’ function in all cases; however, this centrality extends beyond mere bioenergetics. Acting as a metabolic “command hub”, mitochondria orchestrate the delicate balance of redox signaling and stemness maintenance, thereby equipping CSCs with the resilience to withstand extreme environmental duress [1,29]. Various factors, such as oxygen, nutrient levels, and oncogenic signaling, which drive CSCs’ plasticity and metabolic switching, support stemness (via redox balance), regulate reactive oxygen species (ROS) levels, and promote survival under stress [30]. It is crucial to consider that ROS functions as a biological double-edged sword: while unchecked surges lead to exhaustion, a calibrated baseline is required to fuel the cell cycle machinery [31].

For a T cell to mount an effective defense, it must undergo rapid clonal expansion. However, this proliferation is strictly gated by oxidative stress; if ROS levels breach a certain threshold, the cell cycle arrests at the G1/S checkpoint to prevent DNA damage [32]. The acquisition of healthy mitochondria (e.g., from MSCs) effectively hits a ‘Redox Reset’ button. By delivering antioxidant enzymes such as Manganese Superoxide Dismutase (MnSOD, or SOD2) and stabilizing the electron transport chain, these donated organelles maintain the T cell within a permissive metabolic window [33]. This ensures that the immune cell avoids premature senescence and sustains the massive proliferation needed to overwhelm the tumor [34]. It is important to look beyond simple energetics to appreciate that mitochondrial transfer restores the delicate ‘Redox Balance’ essential for proliferation.

Importantly, horizontal mitochondrial transfer from MSC to cancer cells rescues respiration-dependent de novo pyrimidine biosynthesis, a process that can also modulate intracellular ROS generation. In the context of tumor–immune interactions, such alterations in ROS levels are known to destabilize the immune synapse, as the specialized interface required for effective T-cell activation and killing. Thus, the same mitochondrial acquisition mechanism that supports cancer cell proliferation may concurrently disrupt ROS-mediated immune synapse integrity, providing a previously underappreciated avenue for immune evasion [35].

Recent findings show single-cell and spatial transcriptomics linking metabolic heterogeneity to immune dysfunction. Recent studies further emphasize that CSCs are critical mediators of immune escape and are highly treatment-resistant [21,36]. Also, some studies show “metabolic hotspots” within tumors where CSCs and immune cells interact [16]. For instance, spatial analysis of head-and-neck cancers revealed that hypoxic, high-glycolysis regions coincide with immunosuppressive signaling (e.g., increased TGF-β and Treg recruitment), creating a specialized, hostile environment that promotes tumor survival and immune escape [17]. Single-cell profiling can identify CSC subpopulations with hybrid glycolytic/OXPHOS states that engage in dysfunctional synapses with T cells or macrophages. This could illuminate how metabolic gradients in the TME shape CSCs and immune crosstalk and highlight potential biomarkers of immune escape (e.g., co-localization of CSC markers with M2 macrophages in hypoxic niches) [18].

Building on these insights, a recent single-cell RNA sequencing (scRNA-seq) study of pancreatic adenocarcinoma (PAAD) has revealed significant metabolic reprogramming of epithelial glycosphingolipids, which serve as a pivotal driver of malignancy, proliferation, and metastasis [37]. As previously reported, the single-cell landscape of pancreatic adenocarcinoma includes a UMAP of 43,580 cells colored by 23 clusters and an annotation of those clusters into 16 major cell types, including cancer stem cells (CSCs), epithelial cells, fibroblasts, and various immune subsets. Utilizing scRNA-seq data from the GSE212966 dataset, encompassing 43,580 cells clustered via Uniform Manifold Approximation and Projection (UMAP) into 16 distinct subgroups, including cancer stem cells (CSCs), epithelial cells, fibroblasts, and various immune populations, the investigation identified elevated glycosphingolipid (GSL) metabolism in tumor-associated epithelial cells, characterized by pathways such as glycolysis, O-glycan biosynthesis, and EMT-related transitions [38].

To bridge the gap between single-cell metabolic findings and clinical practice, a six-gene prognostic risk model was constructed from differentially expressed genes (DEGs) associated with epithelial glycosphingolipid metabolism in pancreatic adenocarcinoma (PAAD) [38]. Using univariate Cox regression, LASSO regression, and multivariate Cox regression, six pivotal genes, *KLK10*, *MT1X*, *LAMA3*, *MET*, *KRT7*, and *SFTA2*, were identified to build a risk score that stratified patients into high- and low-risk groups based on the median value. The high-risk group consistently showed significantly worse overall survival in the TCGA-PAAD training set and two independent validation cohorts (ICGC-PAAD-US and GSE71729), with highly consistent expression patterns of the signature genes across datasets. Additionally, high-risk patients had higher somatic mutation burdens, particularly in key driver genes such as *KRAS* and *TP53*, and exhibited an immunosuppressive tumor microenvironment, characterized by greater infiltration of regulatory T cells and M2-polarized macrophages. This subgroup also exhibited increased sensitivity to specific chemotherapies, such as Acetalax and Selumetinib. For improved clinical utility, a prognostic nomogram was developed by integrating the risk score with clinicopathological variables (age, T stage, N stage, and tumor grade). This nomogram achieved superior predictive performance for 1-, 3-, and 5-year survival, as confirmed by calibration curves and decision curve analysis (DCA) [38,39,40]. Building on these single-cell findings in pancreatic cancer, recent reviews on liver cancer highlight the pivotal role of TME in CSC metabolic rewiring. For instance, TME stimuli such as hypoxia and immune cell interactions drive CSC plasticity, aligning with the metabolic niches and dysfunctional synapses discussed earlier [41].

Despite substantial evidence on metabolic adaptations in liver cancer, the direct interplay between metabolic rewiring of CSCs and tumor microenvironment (TME) stimuli remains underexplored, particularly in cholangiocarcinoma (CCA) [42]. TME-driven metabolic niches shaped by hypoxia, nutrient scarcity, and metabolite accumulation regulate CSC plasticity, promoting shifts toward glycolysis or oxidative phosphorylation to enhance survival and therapy resistance. Moreover, bidirectional CSC-TME crosstalk disrupts the formation of effective CSC–immune synapses, thereby enabling immune evasion [42,43]. Crucially, CSCs actively contribute to immunosuppressive niches by secreting mediators that recruit and polarize immune cells (e.g., macrophages to an M2 phenotype, myeloid-derived suppressor cells, and regulatory T cells) via factors such as CCL2, IL-6, CCL22, VEGF, IL-13, and IL-34. Nutrient deprivation and metabolites like lactate further amplify immunosuppression, impairing effector T cell function and promoting angiogenesis. Disrupting this metabolic-immune crosstalk by inhibiting CSC-derived factors could dismantle protective niches, restore functional immune synapses, and overcome CSC resilience. Targeting TME-modulated metabolic niches alongside immune boosters thus holds promise for more effective combination therapies in liver cancers [44,45,46].

**Figure 2:**
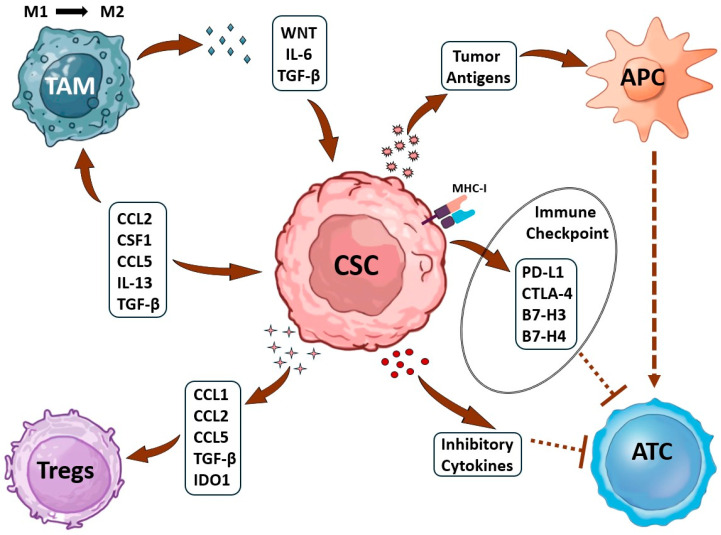
Schematic diagram of the tumor microenvironment showing interactions among immune cells, tumor cells, and signaling molecules. **Shapes:** **Ovals/circles**—Represent cell types: M1 and M2 macrophages, TAM (tumor-associated macrophage), APC (antigen-presenting cell), Tregs (regulatory T cells), *ATC* (activated T cell). **Rectangles/rounded boxes**—Represent soluble factors or membrane-bound molecules: Cytokines: IL-6, TGF-β, IL-13, CSF1 Chemokines: CCL1, CCL2, CCL5 Checkpoint proteins: PD-L1, CTLA-4, B7-H3, B7-H4 Intracellular enzymes: IDO1 Other: WNT (signaling pathway), Antigens, MHC-I, Immune Checkpoint (general), Cytokines (general), Inhibitory (likely inhibitory receptors or signals) **Dashed or dotted boundary** – May denote the tumor mass or the immune-suppressive niche. **Arrows:** **Solid arrows (→)**—Indicate **stimulation**, **activation**, or **secretion** (e.g., tumor cells secreting CSF1 to recruit TAMs; M2 releasing TGF-β to suppress T cells). **Blunted arrows (⊣)** or **T-bars**—Indicate **inhibition** or **suppression** (e.g., PD-L1 binding to PD-1 on T cells; CTLA-4 delivering inhibitory signals; TGF-β suppressing APC function). **Dashed arrows**—May indicate **indirect effects**, **conversion** (e.g., M1 → M2 polarization), or **transcription** (e.g., WNT signaling leading to cytokine expression). **Arrows pointing into a cell** – Represent **receptor-ligand binding** or **uptake** of signals. **Arrows labeled with molecule names**—Indicate the direction of secretion or action (e.g., CCL2 secreted by tumor attracting monocytes) [29].

As illustrated in Figure 2, cancer stem cells act as master conductors, directing the creation of a tumor environment that suppresses the immune system. They achieve this through constant two-way communication with two key accomplices: tumor-associated macrophages and regulatory T cells [47]. In this alliance, both cancer stem cells and macrophages release signaling factors such as CCL2, CSF1, IL-13, and TGF-β that actively push macrophages into a pro-tumor, healing mode (called M2 polarization). In a vicious cycle, these reprogrammed macrophages then send back their own signals, such as WNT, IL-6, and TGF-β, which act as fuel to sustain the cancer cells’ stem-like, resilient properties. To make matters worse, the cancer stem cells deploy a multi-layer defense: they hide their cellular “ID tags” by downregulating MHC-I, display “do not attack” signals such as PD-L1 and CTLA-4, and flood the area with inhibitory cytokines, including CCL2, CCL5, and TGF-β. This combined assault cripples the immune response by disrupting how threats are presented to immune cells, paralyzing the killer T cells, and recruiting more suppressor T cells. The result is a breakdown in the crucial immune attack connection, effectively allowing the tumor to cloak itself and evade destruction [22,48].

## 3. Mitochondrial Transfer by CSCs: Tunneling Nanotubes and Extracellular Vesicles (EVs)

Recent studies show that tumor cells form intercellular conduits (tunneling nanotubes, TNTs; Figure 1) or secrete EVs containing mitochondrial components to relay mitochondria between cells [3,27,49]. For example, mitochondrial transfer occurs across different cells and alters mitochondrial metabolism in recipient cells [4]. Both TNTs and EVs fusion have been shown to carry whole mitochondria or mtDNA to immune cells [50]. Evidence (especially from human tumors) shows that CSCs use these routes to reprogram immune and stromal cells. Studies show that cancer cells can obtain mitochondria from T cells via TNTs to blunt immune attacks, and EVs can deliver mitochondrial content across cells [51]. Remarkably, recent evidence shows that EV-mediated mitochondrial transfer can induce mitophagy in recipient cells: by donating healthy mitochondria, ExMVs trigger clearance of damaged organelles, thereby improving cellular energetics [52,53]. Crucially, this interaction is far from a one-way street; it operates as a distinct ‘parasitic bidirectional exchange.’ Cancer cells do not merely steal healthy mitochondria to revitalize themselves; they actively dump their own damaged, ROS-laden organelles back into the T cells [9]. Direct physical evidence of this mitochondrial appropriation is reported, revealing how cancer cells extend tunneling nanotubes to siphon mitochondria from immune defenders. This act of ‘metabolic theft’ critically drains the T cell’s respiratory capacity, accelerating its exhaustion and paving the way for immune escape [10]. The diverse routes of mitochondrial exchange, including TNT-mediated transfer, EV-mediated delivery, gap junction communication, and paracrine signaling, can be categorized into distinct modes based on donor–recipient pairs, mechanisms, and functional outcomes (Table 1).

Multiple mechanisms of mitochondrial transfer exist: engulfment by a secreted microvesicle, release into the medium followed by endocytosis, or direct shuttling via nanotubes [57,65]. A crucial mechanistic detail often overlooked is the role of Miro1 (Mitochondrial Rho GTPase 1) [55]. Studies have identified this protein as the primary ‘molecular motor’ driving mitochondrial trafficking along the microtubules within TNTs, with Miro1 overexpression directly correlating to the velocity and efficiency of this ‘metabolic hijacking’ [66]. For example, as illustrated below, an exhausted T cell may receive a “sabotaged” mitochondrion from a cancer cell (via TNT or EV) that impairs its function, while a healthy T cell can receive a donated mitochondrion from a mesenchymal stem cell (MSC) that boosts its metabolism [26,67]. Computational tools (such as the mitochondrial-enabled reconstruction of cellular interactions, MERCI) are being developed to quantify these exchanges; high MERCI scores have been linked to poorer clinical outcomes in cancer [7]. In summary, oxidative stress drives TNT formation and exosome release, enabling tumors to rewire immune-cell metabolism [68,69]. Harnessing this knowledge could guide next-generation immunotherapies (e.g., improving CAR-T cell efficacy via mitochondrial donation) and identify circulating biomarkers (e.g., circulating mtDNA, mitochondrial transfer scores) of treatment response [7,70].

## 4. Effects of CSC Mitochondrial Transfer on Immune Cells

Mitochondrial transfer can enhance oxidative metabolism, induce oxidative stress and cause metabolic dysfunction in the recipient cells. This could tip macrophages toward an M2 (tumor-supportive) phenotype and impair T-cell cytotoxicity [44,54]. A Nature study demonstrates that T cells that inherit mutant mitochondria from cancer cells become senescent and exhibit defective effector functions [45]. This process is further exacerbated by the disruption of key metabolic checkpoints that govern T-cell fate. Specifically, the enzymes ATP-citrate lyase (ACLY) and acetyl-CoA carboxylase 1 (ACC1) integrate mitochondrial metabolism with de novo lipogenesis; their dysregulation following aberrant mitochondrial transfer promotes an exhausted T-cell phenotype. In line with this, Somova and colleagues (2025) showed that mesenchymal stem cell-mediated mitochondrial transfer directly regulates B-lymphocyte fate, and similar metabolic rewiring has been implicated in T-cell exhaustion, where impaired ACLY/ACC1 activity reduces the availability of acetyl-CoA for histone acetylation and compromises effector gene expression [71]. Altered metabolism in immune cells often increases PD-L1 expression, further suppressing antitumor immunity (e.g., by stabilizing HIF-1α and PD-L1 in the tumor microenvironment) [48,59,72]. Also, reports show that EV-bound mtDNA activates TLR9/NF-κB in tumor-associated macrophages (TAMs), inducing an M2-like immunosuppressive state (as seen in gastric CSCs models) [56].

## 5. Extracellular Vesicles Cargo and microRNA-Mediated Crosstalk (miRNA/mitomiR Regulation)

CSCs secrete EVs rich in oncogenic microRNAs (mitomiRs) that tune immune and metabolic pathways [5]. Exosomal miRNAs can reprogram multiple immune cells: for example, CSC-derived EVs have been shown to polarize macrophages toward a pro-tumor M2 state and to impair dendritic cell (DC) and T-cell function [6,64]. Notable examples include miR-21 and miR-210. miR-21-carrying exosomes promote macrophage M2 polarization (via RhoB/MAPK) and can upregulate PD-L1 in target cells [11,12]. miR-210, often induced by hypoxia, can rewire recipient cell metabolism and angiogenesis [13]. microRNAs, including miR-21, miR-210, and miR-34a, collectively drive immunosuppression, metabolic rewiring, and therapy resistance. EVs derived from cancer stem cells (CSCs) carry not only mitochondrial components but also a rich cargo of microRNAs (miRNAs) that act as a “metabolic code,” instructing recipient cells to adopt tumor-permissive phenotypes. This code comprises both the *hardware* (mitochondria and mtDNA) and the *regulatory software* (miRNAs), which together ensure that immune cell metabolism is precisely rewired to support tumor progression. To illustrate this, Table 2 summarizes the key CSC-derived EV miRNAs, their verified molecular targets in immune cells, the resulting functional outcomes, and the tumor models in which these interactions have been validated.

The clinical relevance of these EV-miRNA axes is supported by findings that elevated serum levels of CSC-derived EVs carrying miR-21 or miR-210 correlate with poor prognosis, advanced tumor stage, and resistance to immunotherapy [5,6]. Thus, EV-miRNA profiles may serve as liquid biopsies for CSC activity and as predictive biomarkers for response to mitochondrial-targeted or immune-based therapies.

As shown in Table 2, miR-21-containing exosomes promote M2 macrophage polarization via suppression of RhoB and activation of MAPK signaling, while also upregulating PD-L1 in target cells [11,12]. Hypoxia-induced miR-210 delivered by EVs rewires metabolic gene expression (e.g., targeting COX10 and ISCU), leading to mitochondrial dysfunction and increased glycolysis in recipient immune cells [13,73]. The tumor suppressor miR-34a is paradoxically enriched in CSC-derived EVs; it can induce T cell exhaustion by targeting SIRT1 and LAG3 and also suppresses OXPHOS by reducing PGC-1α expression [19,73]. Together, these EV-miRNA cascades create a self-amplifying loop of immunosuppression and metabolic reprogramming that extends beyond the immediate vicinity of CSCs.

Meanwhile, CSCs secrete exosomes containing miRNAs that specifically target immune cells to suppress anti-tumor immunity, creating a spatially and functionally heterogeneous immune response. The spatial and functional heterogeneity of the immune system within the TME is a direct consequence of the mitochondrial and miRNA-based mechanisms CSCs use to hijack and suppress anti-tumor immunity.

Among the key signaling molecules carried by EVs, miRNAs have emerged as pivotal regulators of glucose and lipid metabolism [76,77]. miR-141-3p, a member of the miR-200 family, is widely involved in the progression and metastasis of various cancers [78]. Notably, miR-141-3p is associated with adipose tissue-derived EVs and has been implicated in alleviating obesity-induced hepatic insulin resistance and type 2 diabetes [74]. Additionally, previous studies have reported that phosphatase and homolog may be a target of miR-141-3p [75]. The PTEN gene (Phosphatase and Tensin homolog) is a critical tumor suppressor gene located on chromosome 10q23 and regulates cell growth, survival, and proliferation by antagonizing the PI3K/AKT/mTOR signaling pathway. Loss or mutation of PTEN is common in various cancers, including prostate, breast, glioblastoma, and endometrial cancers. PTEN suppresses downstream signaling cascades to maintain metabolic homeostasis [79,80].

## 6. Ciliary–Mitochondrial Interactions in CSCs: Emerging Organelle Crosstalk

Primary cilia are microtubule-based signaling hubs for pathways such as Hedgehog, Wnt, and PDGF. In cancer stem cells (CSCs), primary cilia are present in a context-dependent manner and have been linked to metabolic adaptation and immune privilege. Recent evidence indicates that cilia physically interfaces with mitochondria through tethering proteins, creating a functional axis that coordinates energy production with extracellular sensing. One such linker is TSGA10, a ciliary–centrosomal protein that binds to mitochondrial complex III (cytochrome c1), facilitating electron transfer and ATP synthesis while limiting ROS generation [81,82]. By positioning mitochondria at the ciliary base, cells can supply ATP directly for ciliary signaling and modulate calcium-dependent responses [83].

Many CSCs retain primary cilia alongside high mitochondrial mass, a feature shared with certain immune-privileged cells (e.g., neurons). Direct evidence linking ciliary–mitochondrial interactions to CSC immune evasion remains limited, and represents a hypothesis-generating framework requiring experimental validation. This ciliary–mitochondrial hub may support CSC survival under metabolic stress and contribute to immune evasion by maintaining redox balance. TSGA10 is overexpressed in several malignancies (melanoma, colon, liver, ovarian cancers) and can suppress HIF-1α nuclear translocation, thereby inhibiting hypoxia-induced glycolysis and angiogenesis [84,85]. In the context of CSC immunometabolism, we hypothesize that TSGA10-mediated coupling represents a vulnerability whereas uncoupling proteins protect ciliated cells from oxidative damage [86]: disrupting this axis (e.g., with ciliogenesis inhibitors or TSGA10-targeting strategies) could collapse CSC metabolic resilience and restore antitumor immunity [83,87].

Cilia-associated proteins recruit mitochondria to the ciliary base to supply ATP or trigger depolarization for calcium signaling [88]. Given that many CSCs possess primary cilia and high mitochondrial content, the “Miro1 hub” supports CSC survival and immune privilege by ensuring immediate energy availability. Targeting this axis (e.g., inhibiting TSGA10 or ciliary signaling) may disrupt CSC immunometabolism [84].

TSGA10 enhances mitochondrial efficiency by promoting ATP production via oxidative phosphorylation while reducing wasteful electron leaks [81,85]. In cancer, TSGA10 exhibits paradoxical behavior: it can act as a tumor suppressor by blocking HIF-1α nuclear entry, inhibiting pro-metastatic genes (*VEGF, MMP2, MMP9*) [89]. Conversely, reduced TSGA10 allows unchecked HIF-1α, encouraging glycolysis, ROS accumulation, and adaptation to hypoxia. This flexibility helps CSCs thrive under stress and resist therapy. By linking ciliary positioning with mitochondrial efficiency and HIF-1α control, TSGA10 emerges as a critical node sustaining CSC energy needs, redox balance, and immune evasion. Disrupting this axis could render CSCs metabolically vulnerable, reducing their ability to reprogram energy pathways [81,90].

Finally, in differentiated thyroid cancer, loss of primary cilia disrupts the ciliary–mitochondrial interaction mediated by VDAC1, leading to mitochondrial fragmentation and apoptosis [87]. In cell lines, ciliogenesis defects reduced ciliation by 80–90%, produced globular fragmented mitochondria, and raised apoptosis to 15–27% (measured by Annexin V/PI and TUNEL), and the effect was largely reversed by the VDAC1 oligomerization inhibitor DIDS [91]. Pharmacological ciliogenesis inhibition (e.g., ciliobrevin A) decreases viability by 40–45%, suggesting that deliberate disruption of this interaction offers a selective therapeutic strategy for cilia-preserving cancers [92,93]. Whether similar effects occur in CSCs remains to be tested, but this evidence supports exploring ciliary–mitochondrial targeting as an orthogonal approach to CSC eradication.

**Figure 3:**
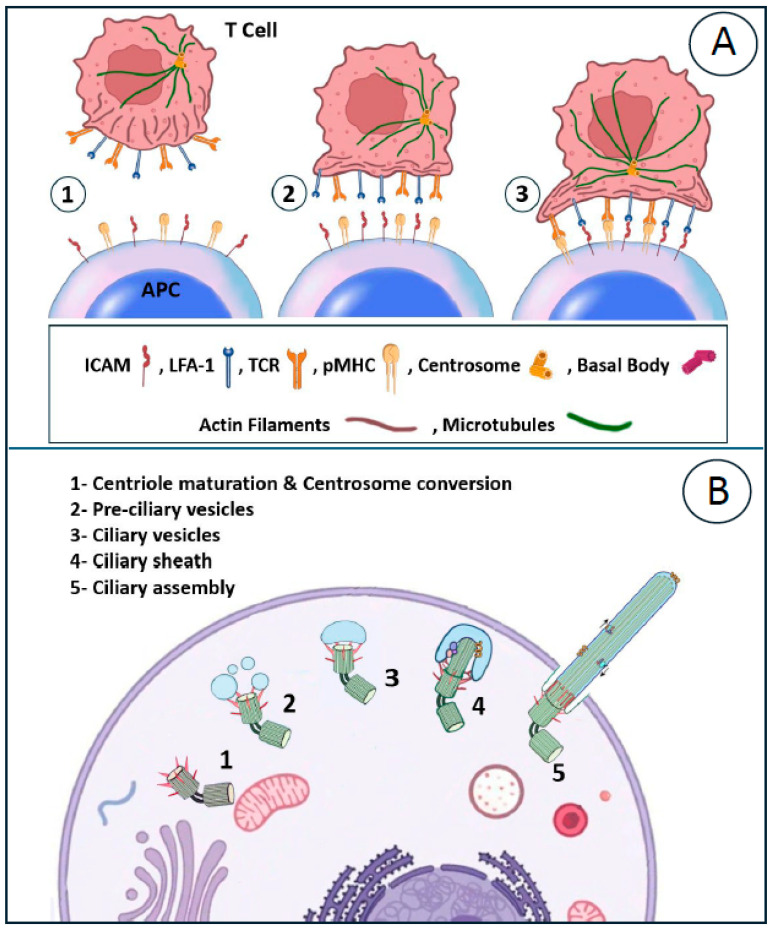
Schematic comparison of key steps in immunological synapse (IS) formation in T cells and primary ciliogenesis. (**A**) IS assembly is triggered by TCR recognition of pMHC on an APC, leading to centrosome polarization, docking at the synaptic membrane, and polarized secretion. Actin initially clusters TCRs in the cSMAC before retracting to the dSMAC, forming a ring around the LFA-1-enriched pSMAC. 1—Centriole maturation and centrosome conversion: The process by which the centrosome matures and converts into a basal body, initiating the formation of a primary cilium-like structure at the T cell–APC interface. 2—Pre-ciliary vesicles: Small vesicles that dock at the distal end of the mature centriole, marking an early step in ciliary assembly. 3—Ciliary vesicles: Larger vesicles formed by the fusion of pre-ciliary vesicles, which elongate to become the ciliary sheath. These three numbered steps correspond to the initial stages of ciliogenesis shown in part B of the figure (steps 1–3) and are integrated with the immunological synapse components (ICAM-1/LFA-1, TCR/pMHC, actin filaments, and microtubules). (**B**) Ciliogenesis is induced by stressors (e.g., serum starvation), involving centrosome-to-basal-body conversion, mother centriole docking with cap vesicle formation, actin redistribution for symmetry breaking, and axoneme nucleation. Both processes feature centrosome docking with local cortical actin clearance, highlighting shared mechanisms adapted for distinct functions [31].

Immune synapses formed by cytotoxic T lymphocytes (CTLs) represent a highly specialized, transient interface that facilitates targeted cell killing during adaptive immune responses. Upon T cell receptor-mediated recognition of antigens on target cells, CTLs rapidly reorganize their cytoskeleton, polarizing the centrosome, the primary microtubule-organizing center, directly to the synaptic plasma membrane. This polarization positions the centrosome as a focal point for directed secretion, where it docks via distal appendages of the mother centriole, creating a secretory domain within the synapse. Lytic granules containing perforin and granzymes traffic along microtubules toward this docked centrosome and undergo polarized exocytosis precisely at the synapse [59]. As illustrated in Figure 3, the formation of the immune synapse in cytotoxic T lymphocytes involves sequential stages from initial T cell APC encounter and stable contact to the mature synapses where centrosome polarization and docking at the plasma membrane creates a focal secretory domain. This mirrors yet functionally diverging from the centrosome-to-basal body conversion and axoneme nucleation seen in primary ciliogenesis [94,95].

Notably, the immunological synapse displays striking morphological and molecular similarities to primary ciliogenesis, while exhibiting a clear functional divergence. In primary cilia, stable docking of the centrosome (the basal body) promotes axonemal outgrowth, enabling sustained environmental sensing. By contrast, the immune system involves brief centrosomal docking, described as a frustrated cilium lacking axoneme extension, thereby repurposing this conserved mechanism for rapid, precisely targeted secretion, which is essential to cytotoxic T lymphocyte function [59].

## 7. Therapeutic Targeting Strategies

Mitochondria in cancer cells have emerged as a highly promising therapeutic target due to the profound metabolic differences between malignant cells and their normal counterparts [96,97]. Unlike healthy cells, tumor cells exhibit a hyperactive metabolic phenotype, preferentially relying on aerobic glycolysis, the hallmark Warburg effect, even when oxygen is abundant, while undergoing extensive metabolic rewiring to survive and proliferate within the nutrient and oxygen-deprived tumor microenvironment. This metabolic plasticity not only drives early carcinogenic events by exacerbating hypoxia but also sustains stemness, metastatic potential, tumor progression, and chemoresistance. Given their central role as the primary energy hub and key regulators of apoptosis, mitochondria have become a focal point for next-generation anticancer strategies [14,15].

Among the most actively investigated agents are mitocans, small-molecule drugs specifically designed to disrupt mitochondrial function in cancer cells, which are currently classified into eight distinct groups based on their molecular targets: Hexokinase 2 (Class I), Bcl-2 family proteins (Class II), Thiol redox systems (Class III), VDAC/ANT (Class IV), the electron transport chain (Class V), the inner mitochondrial membrane (Class VI), the Krebs cycle (Class VII), and mitochondrial DNA (Class VIII) [98,99]. Among mitocans, hexokinase 2 (HK2) stands out as an exceptionally attractive target because it is dramatically overexpressed in most cancers, remains anchored to the outer mitochondrial membrane (facilitating easier access than inner-membrane or matrix targets), and catalyzes the rate-limiting first step of glycolysis while simultaneously inhibiting apoptosis through VDAC blockade [100]. Although numerous HK2 inhibitors (2-deoxy-D-glucose, 3-bromopyruvate, and benserazide) and broader-spectrum mitocans have shown remarkable preclinical efficacy and several have entered clinical trials, their therapeutic success remains limited, largely due to poor specificity and inefficient mitochondrial delivery [101]. Mitochondria produce ATP (adenosine triphosphate) and simultaneously regulate lethal processes, such as apoptosis and necrosis. These lethal functions are primarily regulated by permeabilization of the mitochondrial outer membrane (MOMP), triggered by apoptotic signals and facilitated by the pore-forming activity of proapoptotic proteins, such as the Bcl-2 (B-Cell lymphoma 2) family, Bax (Bcl-2-associated X protein), and Bak (Bcl-2 antagonist/killer protein) [102].

The double-membrane architecture and highly negative membrane potential of mitochondria pose significant barriers to drug penetration, often resulting in off-target effects and suboptimal intracellular concentrations. Consequently, there is an urgent need for advanced, mitochondria-targeted delivery platforms capable of selectively transporting mitocans, particularly HK2-directed agents, directly to the outer mitochondrial membrane of cancer cells, especially the therapy-resistant CSCs subpopulation, thereby maximizing on-target efficacy while minimizing systemic toxicity [103,104].

Multiple therapeutic strategies are emerging to disrupt the CSC–immune metabolic axis, each targeting distinct components of the mitochondrial hijacking and miRNA crosstalk network.

OXPHOS inhibitors—Pharmacological blockade of mitochondrial respiration can deprive CSCs of their preferred energy source. Metformin (complex I inhibitor) and the more selective inhibitor IACS-010759 have shown preclinical efficacy against OXPHOS-dependent CSCs, but clinical development has been limited by toxicity (e.g., neurotoxicity with IACS-010759) [14,15]. Nevertheless, exploiting the metabolic inflexibility of certain CSC subsets remains a viable approach, particularly when combined with glycolysis inhibitors to prevent adaptive switching [2,29].

Blockers of intercellular mitochondrial transfer—Disrupting tunneling nanotube (TNT) formation (e.g., with calpeptin or by targeting Miro1) or inhibiting EV biogenesis (e.g., with GW4869 or nSMase2 knockdown) can prevent both the theft of healthy mitochondria from T cells and the delivery of dysfunctional mitochondria to immune cells [4,9,20]. However, as discussed in Section 8.3, systemic inhibition of TNTs or EVs may cause off-target effects, necessitating CSC-specific delivery approaches.

miRNA antagonists—AntagomiRs against miR-21, miR-210, or miR-34a can reverse CSC-induced immune suppression. For example, miR-21 inhibition restores T cell cytotoxicity and reduces M2 macrophage polarization in preclinical models [11,12]. Clinical translation of anti-miRs faces delivery hurdles (Section 8.2), but lipid nanoparticle formulations and chemical modifications (e.g., LNA-anti-miR-21) are under active investigation [19].

Targeting the ciliary–mitochondrial axis—Given the role of TSGA10 and primary cilia in CSC metabolism, small-molecule ciliogenesis inhibitors (e.g., ciliobrevin A) or TSGA10-disrupting peptides could represent orthogonal therapeutic avenues. In thyroid cancer models, ciliary ablation triggered mitochondrial fragmentation and apoptosis [83,87]; similar strategies might sensitize CSCs to immune attack.

## 8. Translational Challenges and Clinical Barriers

Despite the compelling preclinical rationale for targeting CSC-driven mitochondrial hijacking and miRNA crosstalk, several translational barriers have limited the clinical success of candidate therapeutics. Below we discuss four key challenges: toxicity of OXPHOS inhibitors, delivery hurdles for anti-miRNA agents, tumor specificity of EV pathway inhibitors, and lessons from failed mitochondrial-targeted approaches.

### 8.1. OXPHOS Inhibitor Toxicity

Targeting oxidative phosphorylation (OXPHOS) in CSCs has shown efficacy in preclinical models [14,15], but clinical development has been hampered by on-target toxicity in normal tissues that rely on mitochondrial respiration. Metformin, a mild OXPHOS complex I inhibitor, has favorable safety profile primarily due to its low potency and predominant action in the liver, yet gastrointestinal side effects (diarrhea, nausea) limit tolerability at higher doses [14]. More potent inhibitors have proven problematic. IACS-010759, a highly selective complex I inhibitor that demonstrated robust antitumor activity against OXPHOS-dependent tumors (e.g., AML, glioblastoma), entered Phase I trials but was withdrawn due to dose-limiting neurotoxicity (optic neuritis, peripheral neuropathy) and lactic acidosis [14,15]. This neurotoxicity reflects the vulnerability of retinal ganglion cells and peripheral neurons, which depend heavily on mitochondrial OXPHOS. Similarly, CPI-613 (devimistat), an inhibitor of the tricarboxylic acid (TCA) cycle enzymes pyruvate dehydrogenase and α-ketoglutarate dehydrogenase, has shown only modest activity in pancreatic cancer and biliary tract cancers, with fatigue, nausea, and hyperbilirubinemia as common adverse events [97,99].

These experiences highlight the narrow therapeutic window of systemic OXPHOS inhibition. Strategies to improve tumor specificity include nanoparticle-based mitochondrial targeting [103,104] and prodrug approaches that are activated by the hypoxic TME, but none have yet reached clinical approval.

### 8.2. Anti-miRNA Delivery Challenges

Therapeutic inhibition of oncogenic microRNAs (anti-miRs or antagomiRs) has shown promise in preclinical cancer models, including those targeting CSC-associated miRNAs such as miR-21, miR-210, and miR-34a [12,13,19]. However, successful clinical translation has been hampered by three interrelated challenges: chemical stability, tissue-specific delivery, andcellular uptake.

First, naked RNA oligonucleotides are rapidly degraded by serum nucleases. Chemical modifications, including 2′-O-methyl (2′-OMe), 2′-fluoro (2′-F), locked nucleic acids (LNA), and phosphorothioate backbones, improve stability and binding affinity but can alter toxicity profiles and tissue distribution [19]. Second, efficient delivery to solid tumors (not just the liver, which naturally accumulates nanoparticles) remains suboptimal. Lipid nanoparticle (LNP) formulations, like those used in mRNA vaccines, have been adapted for anti-miRs, but LNP-mediated delivery is largely restricted to the liver, spleen, and bone marrow due to apolipoprotein E-mediated uptake [19,58]. Third, active targeting CSC subpopulations within the tumor mass requires surface ligands (e.g., CD44, CD133 antibodies) conjugated to nanoparticles, adding manufacturing complexity. MRG-106 (cobomarsen) and MRG-201 (remlarsen), summarized in Table 3, are respectively an LNA-based anti-miR-155 oligonucleotide discontinued for hematologic malignancies and a synthetic miR-29a mimic developed for fibrotic diseases that has not advanced for oncology indications.

**Table 3:** Clinical status of leading anti-miR candidates.

Agent	Target	Indication	Clinical Trial Status	Limitation
MRG-106 (cobomarsen)	miR-155	Cutaneous T-cell lymphoma (CTCL), adult T-cell leukemia/lymphoma (ATLL)	Phase II completed; development discontinued	Limited efficacy in solid tumors; proprietary issues [19]
MRG-201 (remlarsen)	miR-29a	Keloid formation (topical)	Phase II completed; not advanced for oncology	Local administration not feasible for visceral tumors

No anti-miR has yet received FDA approval for cancer. The field is now shifting toward small-molecule inhibitors of miRNA biogenesis and miRNA sponge approaches, though these remain preclinical [19,58].

### 8.3. EV Pathway Inhibition and Tumor Specificity

Blocking EV biogenesis or release represents an attractive strategy to disrupt CSC-mediated mitochondrial transfer and miRNA delivery (Section 3 and Section 4) [9,51,56]. EVs carry mitochondrial components (e.g., whole mitochondria, mtDNA, proteins) to recipient cells [9,105]. Their transfer can either enhance radiosensitivity or promote radioresistance in NSCLC, creating a therapeutic controversy [63]. EV cargo profiles (mtO/MtS) may predict radio resistance and metastatic potential [106]. Proposed solutions include using circulating EVs as predictive biomarkers and engineering them to radiosensitize tumors [62,107]. Overall, EVs act both as mediators of therapy resistance and as potential vehicles for mitochondria-based treatments in NSCLC [46,108].

However, EV secretion is a universal cellular process essential for normal physiology, including immune regulation, tissue repair, and neural communication. Thus, systemic EV inhibition risks significant off-target toxicities. The most widely used EV inhibitor, GW4869, targets neutral sphingomyelinase 2 (nSMase2), an enzyme required for ceramide-dependent EV biogenesis. In preclinical models, the candidate agents reduce tumor growth and metastasis [54,62]. However, nSMase2 is also expressed in the central nervous system, where it regulates myelin homeostasis and synaptic function. Chronic GW4869 administration in mice induces neurobehavioral deficits, demyelination, and splenomegaly (due to impaired EV-mediated immune regulation) [9,57]. Other inhibitors, such as calpeptin (blocks TNT formation by inhibiting calpain) and heparin (disrupts EV uptake by competing with surface proteoglycans), lack specificity and affect multiple cellular processes [4,20].

Alternative strategies under investigation include:

• Genetic ablation of EV biogenesis genes (e.g., *Rab27a*, *nSMase2*, *Alix*, *TSG101*) using CRISPR-Cas9 delivered specifically to CSCs (e.g., via anti-CD44 nanoparticles) [62,63].
• Selective targeting of CSC-specific EV cargo loading mechanisms (e.g., inhibition of hnRNPA2B1-mediated miRNA sorting into EVs) [5,58].
• Plasmapheresis or affinity capture of tumor-derived EVs as an extracorporeal strategy (exploratory, not yet clinical) [61].

None of these have advanced to clinical trials. The field awaits small molecules that discriminate between normal and malignant EV secretion.

### 8.4. Failed Approaches: Lessons from Elesclomol and Other Mitochondrial Agents

Several mitochondria-targeted anticancer agents have reached clinical testing but failed because of limited efficacy, toxicity, or poor selectivity, offering important lessons for cancer stem cell (CSC)-directed therapy. Elesclomol, which induces oxidative stress through mitochondrial copper-dependent ROS generation, showed strong preclinical activity but did not improve survival in Phase III metastatic melanoma trials and was associated with serious toxicity; its activity also depended on CTR1/SLC31A1 expression, underscoring the importance of biomarker-based patient selection [14,97,98,99]. Other agents, including gossypol (AT-101), metformin, gamitrinib, Bz-423, MitoVES, and lonidamine, also failed clinically because of insufficient efficacy, toxicity, unstable pharmacokinetics, or limited tumor selectivity [14,15,97,98,99,100,101,102,103]. Collectively, these failures highlight several key challenges for CSC-targeted mitochondrial therapies: global inhibition of mitochondrial function can damage normal tissues, reliable pharmacodynamic biomarkers remain underdeveloped, and CSCs may adapt by switching toward glycolysis when OXPHOS is blocked [7,14,15,22,26,28,29]. Future strategies should therefore emphasize CSC-selective delivery, biomarker-guided patient stratification, and rational combination therapies that simultaneously target mitochondrial metabolism, glycolysis, and resistance pathways [7,26,103,104].

Key lessons from these failures include: (1) inhibiting global mitochondrial function (complex I, electron transport chain) inevitably affects normal proliferating and post-mitotic tissues; (2) pharmacodynamic biomarkers (e.g., circulating mtDNA, mitochondrial ROS) are underdeveloped, making it difficult to confirm target engagement in patients; (3) CSC-enriched tumors may undergo metabolic switching (e.g., upregulating glycolysis when OXPHOS is blocked), necessitating combination strategies; (4) patient stratification based on tumor metabolic phenotype (e.g., high OXPHOS signature, MitoScore) is likely required but not yet standardized [15,28]. Future efforts should focus on CSC-selective mitochondria-targeted delivery (e.g., nanoparticles conjugated with CSC surface antibodies), rational combinations with glycolysis inhibitors (e.g., 2-DG), and robust biomarker-driven trial designs.

## 9. Future Directions

In vivo validation of mitochondrial–EV mechanisms remains limited; most evidence derives from co-culture systems or mouse xenografts with short follow-up. Future research should employ CSC-enriched organoid co-cultures with autologous immune cells, coupled with lineage-tracing and CRISPR screens to identify essential regulators of bidirectional mitochondrial transfer. Additionally, the interplay between mitochondrial hijacking and circadian metabolism (chronometabolism) has not been explored; timing OXPHOS inhibitors to peak CSC mitochondrial activity may improve efficacy while reducing toxicity [109].

### 9.1. Artificial Intelligence and Computational Modeling

The complexity of mitochondrial and EV-mediated communication calls for advanced computational approaches. Deep bioinformatic analyses are already uncovering hidden patterns: for example, gene expression profiling of COVID-19 samples (using tools such as GEO2R/limma) has revealed a surge in mitochondrial respiration and changes in miRNA biogenesis [110]. These large-scale studies can be considered early AI-driven efforts that integrate multi-omics data to identify dysregulated pathways and repurposable drugs [61,110]. In cancer immunology, machine-learning-based image analysis (e.g., single-cell imaging of TNTs) and network reconstruction tools (such as MERCI) could be used to quantify mitochondrial exchanges [111,112,113]. Future AI frameworks might integrate single-cell transcriptomics, proteomics, and spatial imaging to map the “mitochondrial connectome” of tissues. For instance, identifying which T cells have acquired tumor mitochondria (as opposed to those donated by MSCs) could be automated using classifier algorithms trained on multimodal data. Similarly, AI could help predict which miRNAs or EV cargos most strongly influence metabolic reprogramming in recipient cells. In sum, computational AI methods are poised to synthesize data from diverse studies (genomic, imaging, functional assays) to construct predictive models of mitochondrial and metabolic crosstalk [7,110].

### 9.2. Testable Hypothesis: The “Mito-Epigenetic Lock” and Future Viewpoint

Building on these integrated findings, we propose a unifying hypothesis: the ‘Mito-Epigenetic Lock’, in which the transfer of tumor-derived mitochondria into T cells may function as a metabolic “Trojan Horse” within the tumor microenvironment. These hijacked organelles may not merely compromise bioenergetic efficiency but could alter the intracellular availability of key metabolic substrates, including acetyl-CoA and potentially immunomodulatory metabolites such as D-2-hydroxyglutarate (D-2HG), thereby reshaping cellular metabolic signaling. We hypothesize that such metabolic perturbations may influence chromatin remodeling processes and contribute to durable transcriptional reprogramming consistent with T-cell exhaustion phenotypes. Rather than representing a fully irreversible state, this process may establish a persistent epigenetic memory, or “metabolic scar”, that sustains functional impairment even after the initiating tumor-derived signals are reduced. The key predictions and corresponding experimental approaches to test this hypothesis are outlined in Table 4.

Three priorities emerge: (1) In vivo lineage tracing: Use Mito-Cre or mitochondrial barcoding to track tumor-derived mitochondria in immune cells within autochthonous tumor models. (2) CRISPR screens: Genome-scale loss-of-function screens in CSCs co-cultured with T cells to identify regulators of mitochondrial exchange (Miro1, Rab27a, nSMase2). (3) Preclinical combinations: Test whether disrupting mitochondrial hijacking (ciliobrevin A, GW4869, Miro1 inhibitors) synergizes with epigenetic modulators (HDAC inhibitors) and immune checkpoint blockade in organoid-immune co-cultures and humanized mouse models. These efforts will convert the above-mentioned Mito-Epigenetic Lock hypothesis into clinically actionable strategies that eradicate CSCs by dismantling their metabolic-immune control system.

## 10. Conclusions

This review synthesizes the evidence establishing mitochondria as intercellular messengers in CSC immune evasion. The evidence reviewed here establishes that mitochondria are not isolated intracellular powerhouses but function as dynamic intercellular messengers within the tumor microenvironment. Cancer stem cells (CSCs) exploit at least three distinct routes of mitochondrial communication, tunneling nanotubes (TNTs), extracellular vesicles (EVs), and gap junctions, to reprogram immune cell metabolism and enforce immune privilege. Key findings include: (i) CSCs siphon functional mitochondria from cytotoxic T lymphocytes via TNTs, accelerating T cell exhaustion and impairing antitumor immunity [9,10]; (ii) CSC-derived EVs deliver dysfunctional mitochondrial DNA and oncogenic microRNAs (e.g., miR-21, miR-210, miR-34a) to tumor-associated macrophages, driving M2 polarization and further immunosuppression [11,12,56]; (iii) metabolic plasticity enables CSCs to switch between glycolysis and OXPHOS, and mitochondrial hijacking stabilizes HIF-1α and upregulates PD-L1 expression [48,59]; and (iv) single-cell and spatial transcriptomics reveal “metabolic hotspots” where CSCs form dysfunctional immune synapses, correlating with poor prognosis [16,18].

This integrated perspective explains why many current immunotherapies achieve only transient responses: they target the heterogeneous tumor mass but fail to disrupt the CSC-driven communication grid that sustains immune suppression. Effective treatment must address the root cause, CSC plasticity and its mitochondrial–miRNA regulatory networks, rather than solely attacking downstream manifestations (the “branches” instead of the “root”). From a therapeutic perspective, these considerations suggest that interventions targeting mitochondrial transfer alone may be necessary but not sufficient to fully restore immune competence; for example, through disruption of intercellular transport pathways mediated by proteins such as Miro1 or by inhibiting tunneling nanotubes and extracellular vesicle trafficking. A more effective strategy may involve a combinatorial or “dual-hit” approach that simultaneously limits pathological mitochondrial exchange and modulates epigenetic regulatory circuits to reprogram exhausted immune cells and restore antitumor immunity.

## Figures and Tables

**Table 1:** Modes of Mitochondrial Communication in the Tumor Microenvironment.

Mode	Donor Cell	Recipient Cell	Mechanism	Functional Outcome
TNT-mediated whole-organelle transfer	Cancer cells (including CSCs)	Cytotoxic T lymphocytes (CTLs), macrophages	Tunneling nanotubes (TNTs) form intercellular conduits; Miro1 drives mitochondrial trafficking along microtubules; cancer cells siphon healthy mitochondria from T cells or transfer damaged, ROS-generating mitochondria back to immune cells [9,10,20]	T cell exhaustion (reduced respiratory capacity, accelerated senescence); impaired cytotoxic function; macrophage polarization toward M2-like phenotype [44,54]
TNT-mediated whole-organelle transfer	Mesenchymal stem cells (MSCs)	T cells, damaged cancer cells	MSCs donate functional mitochondria via TNTs; Miro1-dependent transport delivers healthy organelles with antioxidant enzymes (e.g., MnSOD/SOD2) [33,55]	Revitalization of T cell metabolism; restoration of redox balance; rescue of exhausted immune cells; enhanced antitumor immunity [7,26]
EV-mediated transfer of mitochondrial components	Gastric CSCs, other cancer cells	Tumor-associated macrophages (TAMs), T cells, stromal cells	EVs carrying dysfunctional mitochondrial DNA (mtDNA), whole mitochondria (mito-exosomes), or mitochondrial proteins are released and taken up by recipient cells via endocytosis or fusion [56,57,58]	TLR9/NF-κB activation in TAMs drives M2 immunosuppressive polarization; disruption of OXPHOS; shift toward glycolysis; elevated PD-L1 expression; immune evasion [48,56,59]
EV-mediated transfer of mitochondrial components	Cancer cells, MSCs	Cancer cells (including CSCs), immune cells	EVs deliver antioxidant capacity via functional mitochondria; EV surface integrins determine tissue/cell targeting specificity [60,61]	Radiation resistance (mitochondria act as “metabolic shields” neutralizing ROS); enhanced tumor survival; potential radiosensitization depending on context [62,63]
Gap junction-mediated transfer	Stromal cells (e.g., MSCs, fibroblasts)	Cancer cells, immune cells	Connexin 43 (Cx43)-formed gap junctions allow direct cytoplasmic continuity; small molecules and mitochondria-derived vesicles can pass between adjacent cells [23,49]	Metabolic coupling; transfer of calcium signals and small metabolites; potential for direct organelle exchange (less characterized than TNTs/EVs) [8]
Secreted factor-induced metabolic reprogramming	CSCs, MSCs, TAMs	Immune cells (T cells, macrophages, DCs), CSCs	Paracrine signaling via cytokines (IL-6, IL-8, TGF-β, CCL2, CCL22, VEGF) and metabolites (lactate) alters recipient cell metabolism without direct organelle transfer [46,47,64]	Induction of glycolysis in TAMs; recruitment of regulatory T cells; suppression of effector T cell function; stabilization of HIF-1α; reinforcement of CSC stemness and immune privilege [43,44,48]
Cell fusion-mediated transfer	Cancer cells	Immune cells, stromal cells	Spontaneous fusion between cancer cells and immune cells (e.g., macrophages) generates hybrid cells containing mixed mitochondrial populations [26]	Acquisition of stem-like properties; transfer of mitochondrial DNA haplotypes; potential for immune evasion via chimeric antigen presentation (mechanistically less defined in humans) [25,26]

**Table 2:** Key CSC-derived EV miRNAs, immune cell targets, and functional outcomes.

miRNA	EV Source	Verified Target(s) in Immune Cells	Functional Outcome	Tumor Model(s)	Refs.
miR-21	Breast, colon, glioblastoma CSCs	RhoB, PTEN, PDCD4	M2 macrophage polarization; increased PD-L1; impaired T cell cytotoxicity	Mouse xenograft, co-culture	[11,12]
miR-210	Hypoxic CSCs (various)	COX10, ISCU, RAD52	Metabolic shift to glycolysis; mitochondrial ROS elevation; T cell exhaustion	Pancreatic, breast, glioma	[13,73]
miR-34a	Liver, pancreatic CSCs	SIRT1, LAG3, PGC-1α	T cell senescence; reduced OXPHOS; increased regulatory T cell recruitment	Orthotopic liver model	[19,73]
miR-141-3p	Adipose-derived EVs (obesity)	PTEN, glycogen synthase	Insulin resistance (metabolic indirect); immune modulation not fully defined	NA—primarily metabolic	[74,75]

Note: The miR-141-3p entry is included for completeness as it exemplifies EV-mediated metabolic control; direct evidence in CSC–immune crosstalk is currently limited and requires further investigation.

**Table 4:** Testable predictions and experimental approaches for the Mito-Epigenetic Lock hypothesis. Abbreviations: CSC, cancer stem cell; LC-MS, liquid chromatography-mass spectrometry; ChIP-seq, chromatin immunoprecipitation sequencing; TNT, tunneling nanotube; HDAC, histone deacetylase; ACLY, ATP-citrate lyase; RRBS, reduced representation bisulfite sequencing; IDH, isocitrate dehydrogenase.

Prediction	Experimental Approach
T cells receiving CSC mitochondria show increased acetyl-CoA and histone hyperacetylation at exhaustion loci (*Pdcd1*, *Lag3*, *Havcr2*)	Co-culture CSCs with naïve CD8^+^ T cells; isolate T cells post-transfer; measure acetyl-CoA by LC-MS; perform ChIP-seq for H3K27ac/H3K9ac; assess exhaustion markers by flow cytometry
Inhibiting mitochondrial transfer (Miro1 knockdown, TNT disruption) prevents the lock and preserves T cell function	Use Miro1 siRNA in CSCs or T cells; quantify transfer via MitoTracker; evaluate T cell proliferation, cytotoxicity, and exhaustion in vitro and in murine models
Epigenetic modulators reverse exhaustion after transfer has occurred	Treat mitochondrial-recipient T cells with HDAC inhibitors (entinostat) or ACLY inhibitors (BMS-303141); assess rescue of effector function
D-2HG from CSC mitochondria cause DNA hypermethylation at effector genes	Measure D-2HG by LC-MS; perform RRBS; compare IDH mutant vs. wild-type CSCs

## Data Availability

No new data were created or analyzed in this study. Data sharing is not applicable to this article.
